# Detection of HIV‐1 Antibodies in Saliva of Persons Living With HIV Using Blood‐Based First Response HIV 1‐2.O Card Test

**DOI:** 10.1002/jcla.70069

**Published:** 2025-06-12

**Authors:** Enoch Aninagyei, Comfort Addo Boatey, Gifty Larbi, Wilson Bright Tsidi, Raphael Eyram Amemo, Ebenezer Tawiah Nyarkotey, Godknows Afenya, Desmond Omane Acheampong

**Affiliations:** ^1^ Department of Biomedical Sciences, School of Basic and Biomedical Sciences University of Health and Allied Sciences Ho Volta Region Ghana; ^2^ Department of Biomedical Sciences, School of Allied Health Sciences University of Cape Coast Ghana; ^3^ Laboratory Department Catholic Hospital, Anfoega, Catholic Health Service Trust‐ Ho Diocese Volta Region Ghana

**Keywords:** HIV‐1 antibodies, human immunodeficiency virus, opportunistic infections, saliva samples, self‐Lollisponge device, sHIV‐1 ab, stomatorrhagia

## Abstract

**Background:**

This study tested HIV‐1 antibodies in saliva samples (sHIV‐1 Ab) collected by the Self‐Lollisponge device.

**Methods:**

Blood and saliva from confirmed persons with HIV and HIV‐negative controls were analyzed for HIV‐1/2 antibodies using the blood‐based First Response HIV 1‐2.O Card Test. The sampling device containing sHIV‐1 Ab was stored at 6°C for 60 days, with intermittent testing on days 2, 5, 10, 20, 30, and 60. Regression analysis was done to assess the relationship between the presence of sHIV‐1 Ab and independent variables.

**Results:**

The sensitivity and the specificity of detecting sHIV‐1 Ab were 72.9% (95% CI: 63.92%–80.65%) and 100% (95% CI: 92.89%–100.00%), respectively. The presence of opportunistic infections (AOR = 13.1, *p* < 0.001), having stomatorrhagia (AOR = 4.56, *p* = 0.0022), and hyperviremia (> 201 copies/mL) (AOR = 4.91, *p* = 0.0225) heightened sHIV‐1 Ab detection. Furthermore, fatigue (AOR = 12.1, *p* = 0.0024), fever (AOR = 3.5, *p* = 0.0144), and weight loss (AOR = 10.9, *p* = 0.0318) increased the odds of having sHIV‐1 Ab in persons living with HIV (PLWHIV). sHIV‐1 Ab was identified in over 90% of PLWHIV with opportunistic infections (OIs) and stomatorrhagia, OIs and hyperviremia, and stomatorrhagia and hyperviremia. Upon storage for 60 days, the sHIV‐1 Ab was detected in all the samples.

**Conclusion:**

Saliva could be an alternative to blood for diagnosing HIV. In addition, the Self‐Lollisponge device was found to be user‐friendly, acquiescent to all settings, and cheap, and can preserve sHIV‐1 Ab for at least 60 days.

## Graphical abstract

- The sensitivity of detecting HIV‐1 antibodies in saliva samples collected with the Self‐Lollisponge device was 72.9% (86/118).
- Detection of HIV‐1 antibodies in saliva depended on the presence of opportunistic infections, stomatorrhagia, and abnormal viral load (> 75 copies/mL)
- The Self‐Lollisponge device was found to be user‐friendly, acquiescent to all settings and patients.

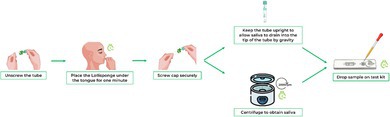

## 1 Introduction

Human immunodeficiency virus (HIV) infection is an immunosuppressive infection caused either by HIV‐1 or HIV‐2 [1]. HIV‐1 is known to cause global infections [2] while HIV‐2 is limited to West Africa [1], though minimal cases have been found in other parts of the world, such as other parts of Africa, Europe, India, and the United States [1]. However, in Ghana, HIV‐1 infections predominate [3] which is tested and confirmed in two stages. First, a blood‐based first‐line test and secondly, confirmed using the OraQuick test kit [4]. The OraQuick uses oral fluids [5], collected by rubbing the sample collection kit around the gum, at the root of the teeth into the gum. This procedure causes trauma to the patients, especially children with weak gums and patients with fulminant HIV, coupled with oral thrush, where the gum is easily injured. This traumatic procedure could lead to auto‐transmission of buccal pathogens through the site of injury. This is common in resource‐limited settings [6]. Nevertheless, the possibility of using oral fluids to detect HIV antibodies, in the case of the OraQuick confirmatory test, makes it worthwhile exploring the use of saliva for the detection of HIV antibodies as a first‐line test, eliminating the need for blood sampling and traumatized gums in HIV testing. It is for this reason that the Self‐Lollisponge device was used to collect saliva samples for HIV antibody testing. Through experience, the sample device has been found to collect adequate salivary samples for laboratory testing. Another advantage of the Self‐Lollisponge device is that it induces saliva production in the mouth within a few seconds because of the lemon‐aromatized nature of the saliva‐collecting cap. Some researchers have attempted to detect HIV antibodies in saliva with some level of success [7, 8]. Balamane et al. detected HIV RNA in saliva [7]. Although Webber et al. detected HIV antibodies, the study used Hema‐Strip HIV‐1/2, Sero‐Strip HIV‐1/2, and Saliva‐Strip HIV‐1/2. Therefore, the First Response HIV‐1/2.O test kit is yet to be evaluated on salivary samples. In both studies [7, 8], participants were asked to provide 5–20 mL of saliva using a sputum container. This practice presents a biohazardous risk as it could facilitate the transmission of the virus to the healthcare practitioner if the container is contaminated with the sample. The Self‐Lollisponge device is a self‐sampling device that is placed in the mouth to collect saliva samples. This sampling procedure can be applied to both confined and ambulatory patients.

To achieve the UNAIDS 95–95‐95 target by the end of 2025, the Ghana Health Service, on July 24, 2023, launched a self‐testing strategy to encourage Ghanaians to know their HIV statuses [9]. Four test kits have undergone evaluation studies in Ghana for the HIV self‐testing program. They were the First Response self‐test kit, OraQuick, Mylan, and Biosure [10]. Except for the OraQuick testing device, which is mostly limited in supply in Ghana [11], the other kits are blood‐dependent. Self‐collection of blood samples could make the HIV self‐testing program unsuccessful. The First Response HIV 1‐2.O Card Test is commonly used as the first‐line test for HIV diagnosis in Ghana; however, the test kit has not been explored on saliva samples. Therefore, this study assessed the efficiency of the First Response HIV 1‐2.O Card Test on saliva samples as a non‐invasive sample for HIV self‐testing.

## 2 Materials and Methods

### 2.1 Study Design, Participants, and Sites

This cross‐sectional study selected participants with HIV from two health facilities in Ghana. The facilities were Ngleshie‐Amanfrom in the Greater Accra region and Catholic Hospital, Anfoega, in the Volta region. These facilities were selected due to their track records of achieving high viral suppression and desirable ART‐compliant rates in persons living with HIV (PLWHIV).

### 2.2 Number of Participants Studied and Selection Criteria

One hundred and eighteen (118) PLWHIV were conveniently selected, with prior written consent. By these selection criteria, a PLWHIV who consented to take part in the study was selected. PLWHIV were selected from the ART Clinics during routine check‐ups. Participants were selected from July to September 2024. The ART Clinic Nurse was trained to seek prior informed consent. To evaluate the specificity of the sHIV Ab detection, saliva samples were collected from 50 patients without HIV antibodies; 30 of the 50 HIV‐negative samples came from individuals with malaria, typhoid, and hepatitis B.

### 2.3 Selection Criteria

The key inclusion criterion was participant testing positive by both first‐line and confirmatory tests, with a previous detectable viral load of any quantity. In addition, the participant should have HIV Ab detected in their blood samples.

### 2.4 Study Variables

Variables such as age, gender, viral load, clinical presentation, stomatorrhagia, and the presence of opportunistic infections were retrieved from the patient's records. Furthermore, the ART regimen was also recorded.

### 2.5 Blood and Saliva Samples Collection

**FIGURE 1:**
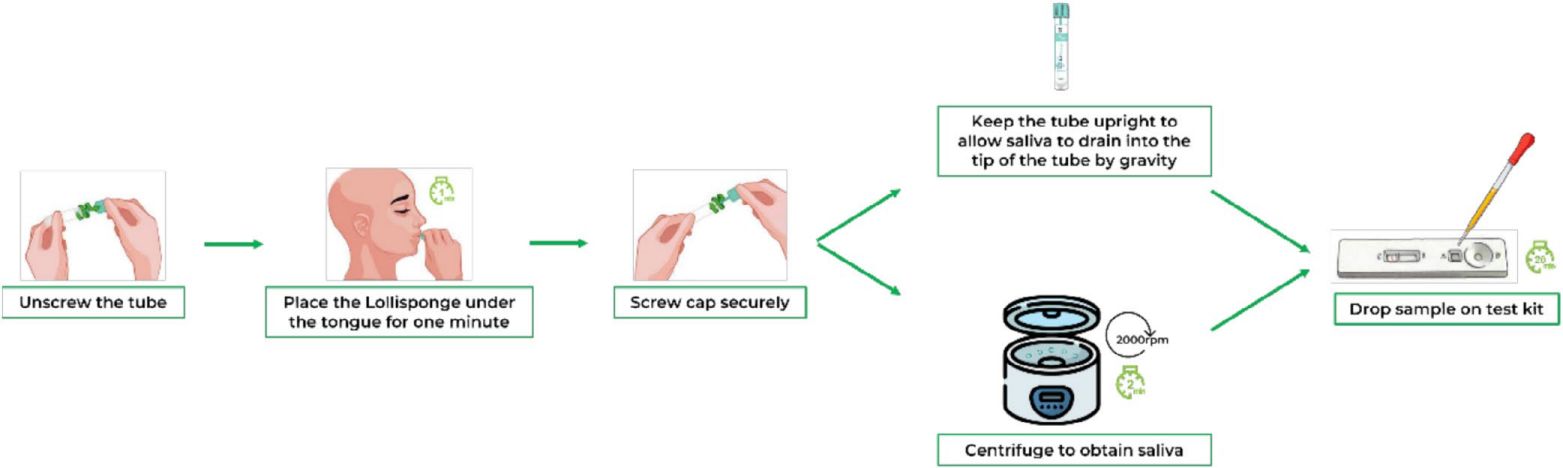
Sequence of the use of the Self‐Lollisponge device for saliva sample collection, processing, and testing.

No scheduled appointment was made for sample collection. Samples were collected when the participants visited the health facility. On the day of participant selection, the phlebotomist collected 4 mL of whole blood into an EDTA tube. Subsequently, the participants or guardians self‐collected the saliva samples from the phlebotomy desk, using the Self‐Lollisponge device, as shown in Figure 1. After this, the blood and the saliva samples were labeled with the participants' numbers.

### 2.6 Detection of HIV 1‐2.O Antibodies From Blood and Saliva Samples

HIV‐1 Ab or sHIV‐1 Ab was detected from both the blood and saliva samples using the blood‐based First Response HIV 1‐2.O Card Test (Premier Medical Corporation Private Limited, Gujarat, India). In brief, 20 μL of either blood or saliva was dispensed into the appropriate sample window, and immediately, one drop of the test buffer was added. The result was read between 15 and 25 min after adding the test buffer.

### 2.7 Assessing the Stability of the HIV Antibodies in the Self‐Lollisponge Device

The Self‐Lollisponge device containing saliva samples that tested positive for HIV Ab was kept at 6°C for 60 days, with intermittent testing on days 2, 5, 10, 20, 30, and 60. On each follow‐up day, the Self‐Lollisponge device was brought to room temperature (25°C), unscrewed, and 20 μL of saliva was collected to perform the test. If adequate saliva was not obtained, the Self‐Lollisponge device was spun at 2000 rpm for 2 min to get enough saliva to perform the test (Figure 1). The Self‐Lollisponge device was immediately returned to the fridge.

### 2.8 Data Analysis

A Chi‐Square test was used to determine the association between saliva HIV antibody test outcome and independent variables. Subsequently, bivariate or multivariate regression analysis was done to assess the relationship between the variables.

## 3 Results

**FIGURE 2:**
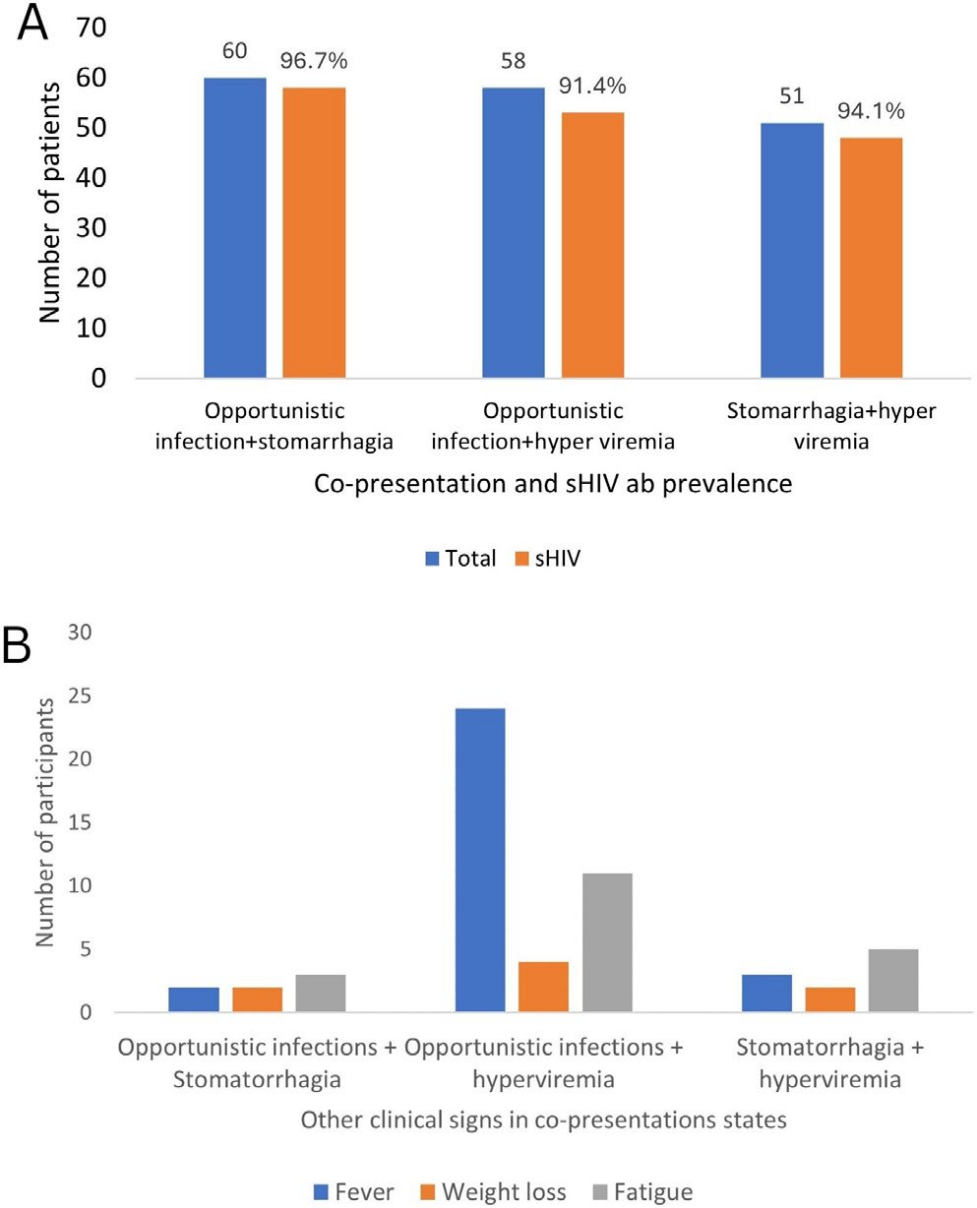
Prevalence of sHIV‐1 Ab (A) and association of other clinical signs (B) among PLWHIV with co‐presentations.

The sensitivity and the specificity of detecting sHIV‐1 Ab were 72.9% (86/118) (95% CI: 63.92%–80.65%) and 100% (50/50) (95% CI: 92.89%–100.00%). When tested for HIV Ab, all the saliva samples collected from the patients without HIV and the patients with malaria, typhoid, and hepatitis B were negative. Therefore, the positive and the negative predictive values were 100.00% (95% CI: 95.80%–100.00%) and 60.98% (53.76%–67.75%), respectively. No HIV‐2 antibodies were detected in either the blood or saliva samples. The distribution of the persons with detectable sHIV‐1 Ab was similar between study sites (*p* = 0.233), gender (*p* = 0.370), and across age groups (*p* = 0.577) (Table 1). Sixty PLWHIV presented with opportunistic infections, out of which 93.3% (54/60) had sHIV‐1 Ab detected. The odds of detecting sHIV‐1 Ab in PLWHIV having opportunistic infections were 13.1 (95% CI: 4.2–40.8, *p* < 0.001) compared to PLWHIV without opportunistic infections. Further, having stomatorrhagia was associated with sHIV‐1 Ab detection, compared to those without stomatorrhagia (AOR = 4.56, 95% CI: 1.7–12.1, *p* = 0.0022). In addition, having moderate copies of viral load counts was associated with sHIV‐1 Ab detection, compared to having very low viral load count (AOR = 4.91, 95% CI: 1.3–19.5, *p* = 0.0225). Finally, the odds of detecting sHIV‐1 Ab significantly increased in PLWHIV presenting with fatigue (AOR = 12.1, 95% CI: 2.4–61.1, *p* = 0.0024), fever (AOR = 3.5, 95% CI: 1.3–9.7, *p* = 0.0144) and weight loss (AOR = 10.9, 95% CI: 95% CI: 1.2–97.0, *p* = 0.0318), compared to PLWHIV with viral suppression who only reported for routine check‐up and drug refill (Table 2). When co‐clinical presentations were analyzed, 60 PLWHIV exhibited both opportunistic infections and stomatorrhagia, of which 96.7% had sHIV‐1 Ab detected. Additionally, both opportunistic infections and hyperviremia were seen in 58 PLWHIV, out of which 91.4% tested positive for sHIV‐1 Ab. Furthermore, 51 PLWHIV presented with stomatorrhagia and hyperviremia, of which 94.1% had sHIV‐1 Ab detected (Figure 2A). Furthermore, it was observed that fever and fatigue were associated with the co‐presence of opportunistic infections and hyperviremia (Figure 2B).

**TABLE 1:** Association of demographic details of the study participants with saliva HIV‐1 antibody status.

Variable	Total *N* (%)	Saliva HIV antibodies		
		Positive *N* (%)	Negative *N* (%)	*p*
Study site				0.233
Catholic Hospital, Anfoega	45 (38.1)	30 (66.7)	15 (33.3)	
Ngleshie‐Amanfrom Polyclinic	73 (61.9)	56 (76.7)	17 (23.3)	
Age range (yrs)				0.577
0–9	6 (5.1)	3 (50)	3 (50)	
10–19	8 (6.8)	7 (87.5)	1 (12.5)	
20–29	17 (14.4)	12 (70.6)	5 (29.4)	
30–39	24 (20.3)	19 (79.2)	5 (20.8)	
40–49	32 (27.1)	25 (78.1)	7 (21.9)	
50–59	24 (20.3)	15 (62.5)	9 (37.5)	
≥ 60	7 (5.9)	5 (71.4)	2 (28.6)	
Gender				0.370
Male	29 (24.6)	23 (79.3)	6 (20.7)	
Female	89 (75.4)	63 (70.8)	26 (29.2)	

**TABLE 2:** Association of clinical details of the study participants with saliva HIV‐1 antibody status.

Variable	Total *N* (%)	Saliva HIV Abs		*X* ^2^ (*p*)	AOR (95% CI)
		Positive *N* (%)	Negative *N* (%)		
Opportunistic infections				< 0.001	
Present	60 (50.8)	56 (93.3)	4 (6.7)	< 0.001	13.1 (4.2–40.8)
Absent	58 (49.2)	30 (51.7)	28 (48.3)		Ref
Stomatorrhagia				0.001	
Present	96 (81.4)	76 (79.2)	20 (20.8)	0.0022	4.56 (1.7–12.1)
Absent	22 (10)	10 (45.5)	12 (54.5)		Ref
Viral load (copies/mL)				< 0.001	
Moderate (> 201copies)	58 (49.2)	54 (93.1)	4 (6.9)	0.022	6.07 (1.29–28.49)
Low‐level (76–200 copies)	15 (12.7)	11 (73)	4 (27)	0.098	3.75 (0.78–17.99)
Undetectable	33 (27.9)	14 (42.4)	19 (57.6)	0.876	0.90 (0.25–3.29)
Very low‐level (20–75 copies)	12 (10.2)	7 (58.3)	5 (41.7)		Ref
Presenting symptoms				0.039	
Fatigue	22 (18.6)	20 (90.9)	2 (9.1)	0.0024	12.1 (2.4–61.1)
Depression	2 (1.7)	1 (50)	1(50)	0.8941	1.2 (0.07–21.2)
Diarrhea	3 (2.5)	2 (66.7)	1 (33.3)	0.4871	2.4 (0.2–29.7)
Fever	39 (33.1)	29 (74.4)	10 (25.6)	0.0144	3.5 (1.3–9.7)
Weight loss	10 (8.5)	9 (90)	1 (10)	0.0318	10.9 (1.2–97.0)
Lymphadenopathy	2 (1.7)	2 (100)	0		^a^
Musculopapular rash	1 (0.8)	1 (100)	0		^a^
Pharyngitis	3 (2.5)	3 (100)	0		^a^
Anemia	2 (1.7)	2 (100)	0		^a^
Cough	1 (0.8)	1 (100)	0		^a^
Anxiety	1 (0.8)	1 (100)	0		^a^
Skin rashes	1 (0.8)	1 (100)	0		^a^
Routine check‐up	31 (26.3)	14 (45.2)	17 (54.8)		Ref
ART Status^b^					
Compliant	98 (83.1)	70 (71.4)	28 (28.6)	0.431	
Defaulter	20 (16.9)	16 (80)	4 (20)		

^a^ Exaggerated odds ratio due to zero response for that variable.

^b^ Tenofovir‐lamivudine‐dolutegravir.

It was also found that 71.4% of Tenofovir‐lamivudine‐dolutegravir‐compliant PLWHIV and 80% of defaulters had sHIV‐1 Ab detected; however, this did not reach significant differences (*p* = 0.431). Irrespective of the viral load levels, sHIV‐1 Ab was preserved and detected in all the 86 Self‐Lollisponge devices containing sHIV‐1 Ab, which were stored at 6°C for up to 60 days.

## 4 Discussion

In Ghana [12, 13, 14] and elsewhere: Nigeria [15], Ethiopia [16], and South Africa [17], human immunodeficiency virus (HIV) screening is mandatory for all pregnant women. In cases of deteriorating health conditions, HIV screening is advised [18]. In addition, everyone is encouraged to participate in the voluntary testing program for HIV infection [9]. This is why the Ghana Health Service, through the National HIV Control Program, launched the self‐testing program in 2023.

The national protocol for HIV screening in Ghana is a first‐line blood‐based test to be confirmed by the OraQuick buccal swab test in all initial reactive cases [19]. Blood sample collection for laboratory diagnosis has several drawbacks. Some of them are transmission of infections [20], tissue injuries [21], nerve damage [22], and introduction of air embolism into the vessels [23]. In addition, there is difficulty in obtaining blood samples from patients with trypanophobia [24], which is common across all age groups [21].

To avert the challenges of blood sample collection, saliva has been explored as a non‐invasive sample for the detection of infectious pathogens [25, 26, 27, 28]. This study sought to improve self‐testing for HIV by introducing a self‐sampling device called the Self‐Lollisponge device. Overall, the sensitivity of detecting sHIV‐1 Ab was 72.9%. Four factors were identified to have influenced the detection of sHIV‐1 Ab. Thus, the odds of detecting sHIV‐1 Ab were over 13 times in persons with opportunistic infections, over 4 times in patients with stomatorrhagia and patients with higher viral load, and finally, over 12 times in patients with fatigue, 3.5 times in patients with fever, and almost 11 times in PLWHIV who lost weight. These factors may be associated with patients with poorly controlled HIV conditions. This is because PLWHIV presenting with opportunistic infections are severely immunosuppressed [29] with higher viral loads [30]. In such patients, weight loss is very common [31]. Stomatorrhagia could also result from the presence of an oral ulcer with or without oral thrush. When the participant co‐presented with stomatorrhagia, hyperviremia, and opportunistic infections, the rate of detection of sHIV‐1 Ab was over 90%. These conditions are known to be associated with fulminant HIV infection [32]. It was also realized that about 82.1% of the patients without opportunistic infections whose saliva was free from sHIV‐1 Ab were virally suppressed. From the foregoing, it could be concluded that sHIV‐1 Ab detection is associated with poor HIV clinical condition as sHIV‐1 Ab was detected in patients who were virally suppressed. It was interesting to find that 19 out of the 43 virally suppressed (44.2%) patients had opportunistic infections, while 18 out of the 43 patients (41.8%) had stomatorrhagia. This means that opportunistic infections and stomatorrhagia can occur in patients with viral suppression. This conundrum has been previously reported [33], albeit at lower frequencies. Therefore, sHIV‐1 Ab detection could be used as a proxy for assessing treatment efficacy in places where viral load testing is not available.

The blood‐based First Response HIV 1‐2. O Card Test failed to detect sHIV‐1 Ab in 27.1% of PLWHIV with detectable HIV Ab in their blood. Several reasons could be attributed to this observation. The saliva samples may have lower immunoglobulin levels compared to their paired blood samples [34]. It has been reported in patients with SARS‐CoV‐2 that disease antibodies in saliva were about 100 times lower compared to corresponding blood samples [35, 36]. In addition, the timing of the sample collection could also be a factor since random samples may have lower levels of the antibodies compared to early morning samples. This has been proven in a TB/HIV study regarding urinary lipoarabinomannan detection [37]. Furthermore, the testing device was meant for blood sample testing and, for that matter, may be suboptimal for saliva sample testing. Moreover, anti‐HIV inhibitors in saliva could reduce the levels of HIV antibodies in saliva [7, 38].

In previous studies that used saliva‐based test kits, the sensitivity and specificity for HIV saliva strips were 99.4% and 99.4%, respectively [39]. When Salivax kits were used among Indians, the sensitivity and specificity were 100% [40]. Again, in a cross‐sectional study in Malawi, Oral‐fluid‐based HIVST had a sensitivity of 88.9% and a specificity of 98.7% [41]. When the Oraquick Advance rapid HIV‐1/2 test kit was used on saliva instead of buccal swab samples, the sensitivity was 98.03%, while the specificity was 99.68% [42]. Per these data from literature, HIV Ab are better detected when saliva‐based test kits are used. Therefore, blood‐based HIV antibody test kits need further improvement in their sensitivities or limits of detection to be able to perform better on saliva.

The Self‐Lollisponge device was found to be feasible and self‐applicable to collect saliva samples from patients of all ages. The device is also cheap compared to blood sample collection, where several consumables are needed. The instructions to collect the saliva samples were simple to follow since all the participants collected the samples correctly. Therefore, the Self‐Lollisponge device could be used for self‐testing for HIV at any location.

The Self‐Lollisponge device containing the sHIV‐1 Ab was kept in the refrigerator (6°C) for 60 days. It was observed that in all the sampling kits, sHIV‐1 Ab was preserved. One key component of the Self‐Lollisponge device is the aromatized lemon presence in the cap. The presence of this key component will not only stimulate saliva production but could also serve as a protein preservative [43, 44, 45] because it can diffuse from the cap into the sponge. This could be the reason why the saliva samples did not release any offensive smell when stored for that long. The protein‐preservative properties of lemon have been documented in many studies [34, 35, 36].

### 4.1 Conclusion

The Self‐Lollisponge device detected sHIV‐1 Ab in 72.9% of PLWHIV. This study found that saliva could be an alternative sample to blood in the diagnosis of HIV. Therefore, this calls for producing and making available saliva‐based test kits to improve the detection of HIV‐1 Ab in saliva. The detection of sHIV‐1 Ab was associated with a debilitating form of the disease. The Self‐Lollisponge device is non‐invasive, safe, user‐friendly, and acquiescent to all settings and patients. When the samples were stored at 6°C, sHIV‐1 Ab were preserved for up to 60 days.

### 4.2 Limitation

The concentrations of sHIV‐1 Ab in the saliva samples could be lower than in corresponding blood samples; therefore, some samples with sHIV‐1 Ab below the detection limits could be missed. Furthermore, this report did not take into consideration the stage of the disease. Finally, random sample collection, as employed in this study, could lead to false‐negative test outcomes.

## Ethics Statement

The ethical clearance for this study was obtained from the Ghana Health Service Ethics Review Committee (GHS‐ERC: 007/02/22).

## Conflicts of Interest

The authors declare no conflicts of interest.

## Data Availability

The data that support the findings of this study are available from the corresponding author upon reasonable request.
